# Association between frailty and physical function recovery of people who received physiotherapy early rehabilitation during acute hospitalisation: An observational cohort study

**DOI:** 10.1016/j.tjfa.2025.100052

**Published:** 2025-05-16

**Authors:** Jennifer R A Jones, Sue Berney, Chris Michael, Tessa O’Dea, Joleen W Rose, Talia Clohessy, Stacey Haughton, Rebekah McGaw, Cameron Patrick, Mark Hindson, Sharae Theisinger, Elena Gerstman, Rebecca Morris, Lucy Gao, David J Berlowitz

**Affiliations:** a Physiotherapy Department, The University of Melbourne, Parkville, Australia; b Physiotherapy Department, Division of Allied Health, Austin Health, Heidelberg, Australia; c Institute for Breathing and Sleep, Heidelberg, Australia; d Statistical Consulting Centre, The University of Melbourne, Parkville, Australia

**Keywords:** Frailty, Physical function, Rehabilitation, Hospital

## Abstract

**Objectives:**

Examine the effect of frailty on physical function recovery in people admitted to hospital who received a physiotherapy Early Rehabilitation program.

**Methods:**

Observational cohort (1 January 2021 to 31 December 2021). Patients admitted to the acute site at Austin Health, Australia who received an Early Rehabilitation program (targeted physical rehabilitation to address goals aligned to physiotherapy intervention in parallel with acute medical treatment) were eligible. Frailty was measured with the Clinical Frailty Scale (CFS). The primary outcome was, across the CFS, magnitude of change from admission to discharge in physical function assessed with the modified Iowa Level of Assistance Scale (mILOA). Secondary outcomes were length of stay and discharge destination. Generalised additive models were used.

**Results:**

There were 674 patients included in the study. Irrespective of frailty status, mean improvement in physical function from admission to discharge exceeded the minimal datable change of 5.8 points for the mILOA. Larger average improvements in mILOA were observed in patients with lower degrees of frailty (*p* < 0.001 overall effect), where a portion of patients with severe frailty did not make clinically meaningful gains in physical function following Early Rehabilitation. Mean improvement in physical function and predicted probabilities for discharge home were similar; where greater frailty severity was associated with a lesser chance of going home at acute hospital discharge (*p* = 0.002 overall effect).

**Conclusions:**

Validating the link between predictions for change in physical function and discharge home in people receiving early rehabilitation during acute hospitalisation would be of great clinical utility.

## Highlights

• In a cohort over 600 people who received early rehabilitation in hospital.
• On average, physical function improved from admission to discharge.
• However, some severely frail patients did not make clinically meaningful gains; and probability of discharge home declined with increasing frailty.
• Validating the link between physical function and discharge destination is needed.

## Graphical abstract

**Figure:**
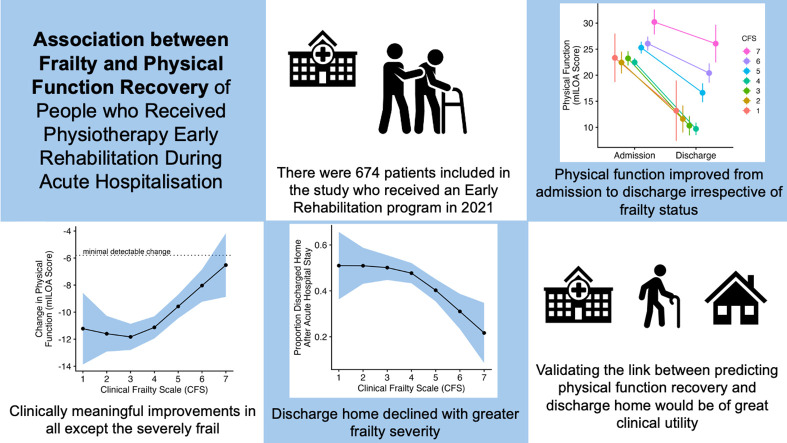

## 1 Introduction

Health systems were designed when single organ illness was the norm and clinical services were organised using a “disease model” [1]. Frailty challenges how modern healthcare is provided as a multidimensional syndrome that is not disease specific. Frailty is characterised by a decline in physiological reserve capacity accompanied by an increased vulnerability to external stressors for example falls and infections [2]. People living with frailty have higher health care utilization, including more frequent and longer hospitalisations, and worse health outcomes than their non-frail peers [3]. Approximately 40 % of adults aged 65 and older admitted to hospital are frail and globally the over 65 age group is projected to double in 25 years [4,5]. It is imperative we accelerate efficient delivery of effective healthcare to address the complexity of ageing and frailty.

People with frailty have greater physical disability, their physical function declines further upon admission to hospital and they take longer to recover than their non-frail peers [6,7]. Exercise and rehabilitation programs are high value, low cost interventions that address physical frailty [[8], [9], [10]]. The Austin Health Physiotherapy workforce in Melbourne, Australia was mobilised in 2014 from a disease-based model to form the Early Rehabilitation service. Physiotherapists of the Early Rehabilitation service provide patients with rehabilitation early in their admission in parallel with acute medical treatment across the hospital in accordance with clinical guidelines [[11], [12], [13], [14]]. Aligning with evidence-based practice, underpinning the Early Rehabilitation service are well-established principles of rehabilitation including a biopsychosocial approach, individualised goal-based therapy and a skilled in-reach team of clinicians [[15], [16], [17], [18]]. The Early Rehabilitation physiotherapists screen for frailty using the Clinical Frailty Scale (CFS), a nine-point scale ranging from fit and healthy to bedridden [19]. The modified Iowa Level of Assistance Scale (mILOA) is a reliable and valid measure responsive to change in acute hospitalised patients and was used to detect change in physical function from beginning to end of the Early Rehabilitation program [20]. The frailty (CFS) and physical function (mILOA) measures are recorded by physiotherapists in the electronic medical record as part of routine clinical practice.

Electronic medical records provide an opportunity to harness rich local data to improve the efficiency and effectiveness of care [21,22]. The CFS is the most common frailty measure used in hospitals and is increasingly being used to assist with prognostication and guide clinical decision making for interventions that may benefit patients [3,23]. There is however, a limited evidence base to support rapid clinical decision making that incorporates frailty, age-related clinical complexity, and physical rehabilitation needs. To address this research gap, the primary aim of the study was to examine the effect of frailty on physical function recovery in a cohort of patients treated by the Early Rehabilitation service. Addressing this research gap would be of great clinical utility in guiding decision making about the physical function recovery potential of patients with frailty and delivery of targeted exercise and physical rehabilitation interventions to accelerate recovery; to ultimately expedite discharge and reduce hospital costs.

## 2 Methods

### 2.1 Study design

An observational cohort study was performed from 1 January 2021 to 31 December 2021. The study was approved by the Austin Health Office for Research (HREC/91913/Austin-2022) and a waiver of consent was provided. The Strengthening the Reporting of Observational Studies in Epidemiology guidelines were followed [24].

### 2.2 Setting

Austin Health is a quaternary metropolitan health service in Victoria, Australia with over 900 beds encompassing acute, aged and rehabilitation services [25]. The Early Rehabilitation service is designed to provide early targeted physical rehabilitation soon after admission to the acute ward. The physiotherapists perform a standardised assessment (includes CFS and mILOA, Fig. 2) to inform prescription of a personalised physical rehabilitation program (e.g. endurance training, or strength training for upper or lower limbs as indicated, or physical function retraining including transfers and ambulation) to address and meet goals aligned to physiotherapy intervention (e.g. mobility or physical function based).

### 2.3 Participants

People admitted to the acute site at Austin Health who were treated by the Early Rehabilitation service four or more times were eligible. If the patient had multiple hospital admissions, the first admission with a CFS recorded was included. People admitted from residential care or who were terminally ill as indicated by a CFS score of nine, were excluded.

### 2.4 Baseline characteristics

Baseline characteristics included age, sex (male or female), socioeconomic status, prior residence (home alone or home with others), admitted via the emergency department (yes or no), admitting specialty, principal diagnosis, frailty (CFS), and physical function (mILOA) at the beginning of the Early Rehabilitation program. Socioeconomic status was derived from census data for residential post codes for the Index of Relative Socioeconomic Disadvantage (deciles from most disadvantaged to least disadvantaged) and the Index of Relative Socioeconomic Advantage and Disadvantage (deciles from most disadvantaged to most advantaged) [26]. The admitting specialty was the medical team the patient was admitted to at the beginning of the hospital admission. Principal diagnoses were classified using the Australian Refined Diagnosis Related Groups (AR-DRGs) using information available in the clinical record at the conclusion of the patient’s episode of care [27,28]. Screening for frailty with the CFS was conducted prospectively by physiotherapists of the Early Rehabilitation service in accordance with published recommendations, as the patients were acutely unwell the CFS was scored based on the two weeks before hospital admission using information from interviewing the patient and their family or caregivers, and the clinical notes [19,29,30]. Physiotherapists of the Early Rehabilitation service completed the online CFS Training Module [31]. Further education (for new and experienced staff) was provided every four months where physiotherapists scored the CFS for clinical vignettes; scores were benchmarked with consistency among raters similar to published studies [32]. Permission to use the CFS was granted by Dr Kenneth Rockwood, Dalhousie University.

### 2.5 Outcome measures

Physical function was assessed with the modified Iowa Level of Assistance scale (mILOA) which assesses the amount of assistance required for six mobility items: 1) lie to sit, 2) sit to stand, 3) walking, 4) negotiation of one step, 5) walking distance and 6) assistive device used. Each item is given a score from zero to six to yield a maximum score of 36, where higher scores indicate greater assistance needed to perform the task and therefore lower levels of physical function [33]. Physical function was assessed with the mILOA after admission to hospital at the commencement of Early Rehabilitation (admission timepoint) and at acute hospital discharge or completion of the Early Rehabilitation program whichever occurred first (discharge timepoint). The mILOA has an established minimal clinically important difference of 5.8 points in acutely hospitalised adults [20].

Acute hospital length of stay was from admission to and discharge from the acute health service. Total hospital length of stay included the subacute inpatient stay if applicable. Discharge destinations were classified as home versus all other categories (residential care, transferred to inpatient rehabilitation at Austin Health or transferred to another health service).

### 2.6 Primary and secondary outcomes

The primary outcome was the magnitude of change (mean difference) in physical function from admission to discharge (i.e. change in mILOA) across frailty scores (i.e. CFS 1 to 7). The secondary outcomes were the proportion of patients discharged home and length of stay for the acute and total hospital discharge timepoints across frailty scores (i.e. CFS 1 to 7). Further, these outcomes were explored comparing patients with (CFS > 4) and without frailty (CFS ≤ 4) using a widely published cut-point [23].

### 2.7 Data collection

**Fig. 1:**
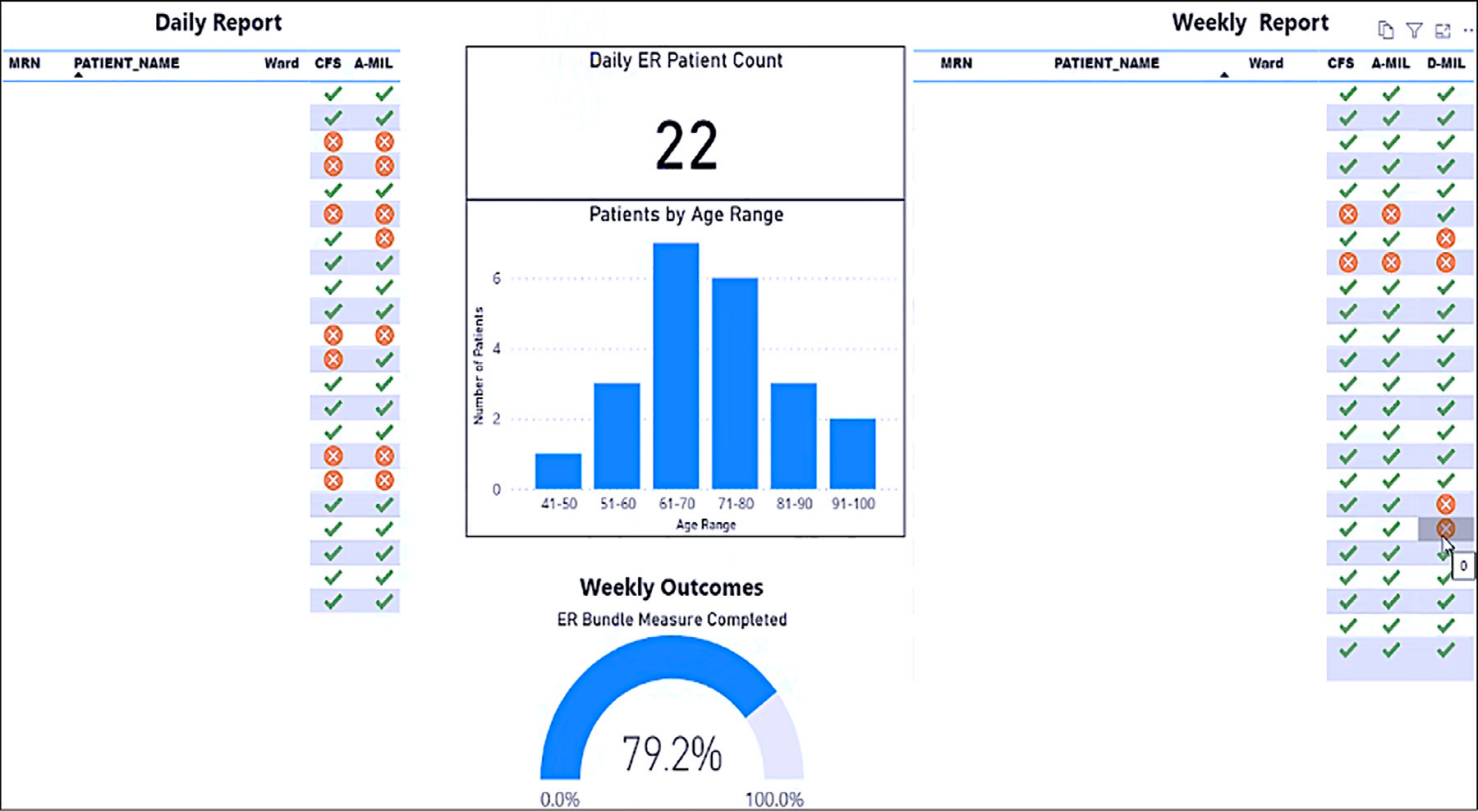
Digital audit with near-real time feedback on completion rates for recording frailty (CFS) and physical function (A-MIL, admission modified Iowa Level of Assistance scale and D-MIL, discharge modified Iowa Level of Assistance scale) in the electronic medical record was provided via a clinical dashboard where a green tick denotes completion and a red cross prompts completion. Abbreviations: A-MIL admission modified Iowa Level of Assistance scale; CFS, Clinical Frailty Scale; D-MIL, discharge modified Iowa Level of Assistance scale; MRN, medical record number.

To support physiotherapists in practice change to assess and record frailty and physical function measures in the electronic medical record the study team employed strategies including: co-development of a digital audit and near-real time feedback clinical dashboard with completion rates for the measures recorded in the electronic medical record (Fig. 1), education and training on how to perform (see 2.4) and then record the measures in the electronic medical record, and clinical champions. Where possible electronic medical record and hospital administrative data were extracted for patient characteristics (e.g. age, sex), clinical care (e.g. frequency of Early Rehabilitation sessions) and outcomes (e.g. discharge destination). Data on prior residence were extracted as free text and manually categorized as home alone or home with others.

### 2.8 Research data

Research data for the study are available via figshare [34].

### 2.9 Data analysis

Data are summarized as median and lower to upper interquartile range [IQR] or mean and 95 % confidence intervals (lower to upper 95 % CI). Patient characteristics were compared across frailty scores (CFS 1 to 7) using the Kruskal-Wallis rank sum test for continuous variables and Pearson’s Chi-squared test for categorical variables.

Generalised additive models (see supplement for more information) were used to assess the average effect of frailty score on each of the outcomes: change in physical function (i.e. change in mILOA score), length of acute hospital stay (days), length of total hospital stay (days), discharge home from acute hospital (yes or no) and discharge home after total hospital stay (yes or no). For change in physical function from admission to discharge, a Gaussian response distribution was used, similar to a standard linear regression model. The length of stay outcomes were modelled as time-to-event using a Cox proportional hazards generalised additive model where a hazard ratio less than one is associated with an increased length of stay and greater than one is associated with a decreased length of stay. For discharge destination, a binomial response distribution and logit link function were used, similar to a standard logistic regression model.

Two models were fitted to each outcome, one treating the exposure as continuous (CFS score 1 to 7) and fitting a smooth nonlinear function, and the other treating the exposure as binary (not frail CFS ≤ 4 vs frail CFS > 4). All models were adjusted for potential confounders: age, sex, socioeconomic disadvantage (Index of Relative Socioeconomic Disadvantage), prior residence, emergency admission and admitting specialty. Continuous confounders were fitted using smooth nonlinear functions.

Patients who survived their acute hospitalisation were analysed for the change in physical function, acute hospital length of stay and discharge destination outcomes (*n* = 674) and those who survived their total hospital admission were analysed for total hospital stay and discharge destination outcomes (*n* = 653).

The Treatment And Reporting of Missing data in Observational Studies (TARMOS) framework informed the analysis of incomplete data [35]. Multiple imputation was used to account for missing frailty and physical function measures and to allow all survivors in the analysis (eTables 1 and 2, see supplement for more information). After accounting for the auxiliary variables in the imputation it was believed the frailty and physical function measures were Missing At Random. eTable 3 shows no association between observed variables and missingness.

A complete case analysis was performed as a sensitivity analysis, i.e. excluding participants with any missing outcome, exposure or confounder variables, and reported in the online supplement. Significance was set at *p* < 0.05. Analyses were performed using R 4.4.2 [36].

## 3 Results

### 3.1 Patient characteristics

**Fig. 2:**
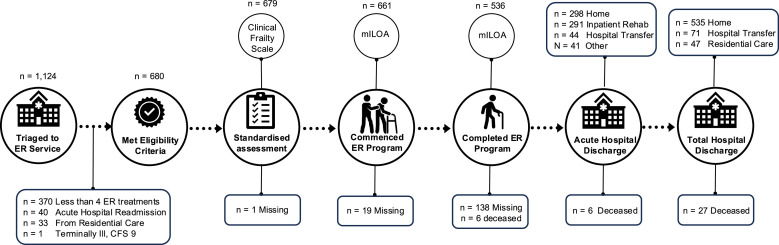
Flow of patients through the study. Abbreviations: ER, Early Rehabilitation.

There were 1124 patients triaged to the Early Rehabilitation service in 2021 and 680 met eligibility criteria for the study (Fig. 2). The cohort median [IQR] age was 75 years [66, 83] with 78 % (*n* = 529) aged 65 years and over (eTable 4). Most were female (*n* = 394, 58 %) and living at home with others (*n* = 468, 69 %; eTable 4). Half were admitted via the emergency department (*n* = 359, 53 %). The most frequent admitting specialties were Orthopaedics (*n* = 408, 60 %), General Medicine (*n* = 98, 14 %), and Stroke, Neurology and Neurosurgery (*n* = 50, 7 %). The most prevalent principal diagnoses were hip or knee replacement (*n* = 254, 37 %), and pelvis, hip or femur trauma or interventions (*n* = 132, 19 %; eTable 4). Age and proportion of females increased with increasing CFS score (Table 1).

**Table 1:** Patient characteristics across the clinical frailty scale.

Characteristic	CFS 1 (*n* = 17)	CFS 2 (*n* = 64)	CFS 3 (*n* = 112)	CFS 4 (*n* = 221)	CFS 5 (*n* = 145)	CFS 6 (*n* = 100)	CFS 7 (*n* = 20)	p-value
Age, years	67 [60, 79]	66 [56, 73]	73 [64, 80]	74 [64, 80]	80 [73, 86]	83 [75, 89]	82 [73, 86]	<0.001
Aged 65 and over	11 (65)	35 (55)	81 (72)	165 (75)	127 (88)	91 (91)	18 (90)	<0.001
Sex								0.002
Female	7 (41)	24 (38)	61 (54)	130 (59)	100 (69)	60 (60)	12 (60)	
Male	10 (59)	40 (63)	51 (46)	91 (41)	45 (31)	40 (40)	8 (40)	
Prior Residence								0.011
Home with others	14 (82)	52 (81)	71 (63)	148 (67)	89 (61)	76 (76)	17 (85)	
Home alone	3 (18)	12 (19)	41 (37)	73 (33)	56 (39)	24 (24)	3 (15)	
Socioeconomic Advantage and Disadvantage	9 [6, 9]	7 [6, 9]	8 [6, 9]	7 [4, 9]	7 [4, 9]	9 [6, 9]	9 [7, 9]	0.2
Socioeconomic Disadvantage	9 [7, 9]	8 [5, 10]	8 [5, 10]	7 [4, 9]	7 [3, 9]	8 [5, 9]	8 [6, 9]	0.4

Continuous data are presented as median [IQR] and categorical data are presented as n (%). The Kruskal-Wallis rank sum test was used for continuous data and Pearsons’ Chi-squared for categorical data. Significance was set at *p* < 0.05. Measures of socioeconomic status were derived from residential post codes for the Index of Relative Socio-Economic Disadvantage and the Index of Relative Socio-Economic Advantage and Disadvantage.

Abbreviations: CFS, Clinical Frailty Scale.

### 3.2 Frailty and physical function measures

There were 679 patients (> 99 %) with a CFS score recorded a median [IQR] of 3 [1, 8] days after admission (Fig. 2). Admission mILOA was measured in 97 % (*n* = 661) of patients and recorded a median of 3 [1, 9] days after admission (Fig. 2). More than 99 % of the patients survived to acute hospital discharge (*n* = 674) where 80 % (*n* = 536) of patients had a discharge mILOA score recorded at a median [IQR] of 8 [4, 18] days after admission (Fig. 2). Admission and discharge mILOA scores were recorded in 78 % of patients (*n* = 529). Patient characteristics (age, sex, prior residence, socioeconomic status, frailty, emergency admission and admitting specialty) were similar for patients with (*n* = 529) or without (*n* = 145) mILOA assessments at both timepoints (eTable 3).

### 3.3 Early rehabilitation program

The median [IQR] number of sessions received during the Early Rehabilitation program was 6 [5, 9] and the number of sessions were statistically different across CFS scores where patients with a CFS of four received the most sessions (8 [6, 10]; eTable 5).

### 3.4 Change in physical function

**Fig. 3:**
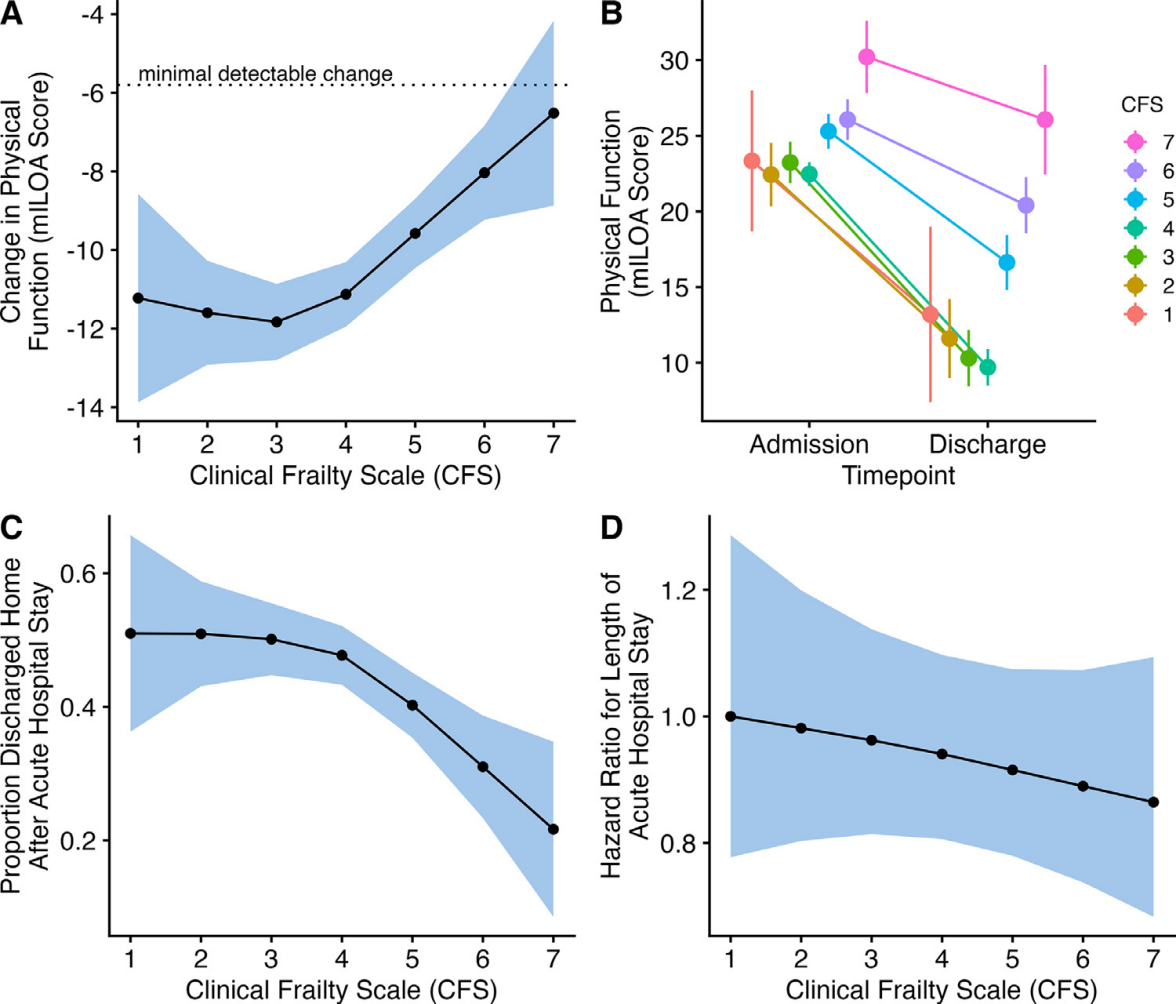
A. Mean difference (95 % CI) across Clinical Frailty Scale scores (CFS 1 to 7) for the change from admission to discharge in physical function as measured with the modified Iowa Level of Assistance (mILOA) scale where the minimal detectable change is 5.8 points. B. Mean (95 % CI) physical function with the modified Iowa Level of Assistance (mILOA) scale at admission (beginning of the Early Rehabilitation program) and discharge from acute hospital (end of the Early Rehabilitation program) across Clinical Frailty Scale scores (CFS 1 to 7). C. Proportion (95 % CI) of patients discharged home after the acute hospital stay across Clinical Frailty Scale scores (CFS 1 to 7). D. Hazard ratio (95 % CI) for total hospital length of stay across Clinical Frailty Scale scores (CFS 1 to 7). Abbreviations: CFS, Clinical Frailty Scale; mILOA, modified Iowa Level of Assistance scale.

Larger average improvements in physical function (mILOA) were observed in patients who were less frail (lower CFS) at admission (*p* < 0.001 overall effect). Irrespective of CFS score, the mean improvement in mILOA score from admission to discharge exceeded the minimal detectable change of 5.8 points (Fig. 3A and 3B; eTable 6). However, there was a portion of patients with severe frailty who did not experience clinically meaningful change in physical function following the Early Rehabilitation program (Fig. 3A). The mean improvement in mILOA for patients without frailty (CFS ≤ 4) was larger at 11.5 (95 % CI: 10.8, 12.2) than in patients with frailty (CFS > 4) who improved by 8.6 (7.7, 9.6), and was significantly different between groups (mean difference 2.9; 95 % CI: 1.6, 4.1; *p* < 0.001).

### 3.5 Acute hospital discharge destination

The acute in-hospital mortality rate was less than one percent (*n* = 6; eTable 1). At acute hospital discharge, 44 % (*n* = 298) went home, 43 % (*n* = 291) were transferred to inpatient rehabilitation at the health service, 7 % (*n* = 44) were transferred as an inpatient to another health service and 6 % (*n* = 41) to other locations (Fig. 2 and eTable 7). Greater admission frailty (across the CFS scores) was associated with a lesser chance of going home at acute hospital discharge (*p* = 0.002 overall effect; Fig. 3C and eTable 8). Patients who were frail (CFS > 4) were 14.7 (7.3, 22.2, *p* < 0.001) percentage points less likely to return home, with the model estimating 35.0 % (29.4, 40.6) of patients with frailty returning home compared to 49.8 % (45.5, 54.0) for patients who were not frail.

### 3.6 Acute hospital length of stay

The median [IQR] acute hospital length of stay for the cohort was 9 [5, 21] days (eTable 9). The relationship between frailty score and acute hospital length of stay was not statistically significant (*p* = 0.490; Fig. 3D and eTable 10). Comparing patients who were not and were frail, there was no statistically significant difference in acute hospital stay (HR = 0.86; 95 % CI: 0.73, 1.02; *p* = 0.090).

### 3.7 Discharge destination after total hospital stay

**Fig. 4:**
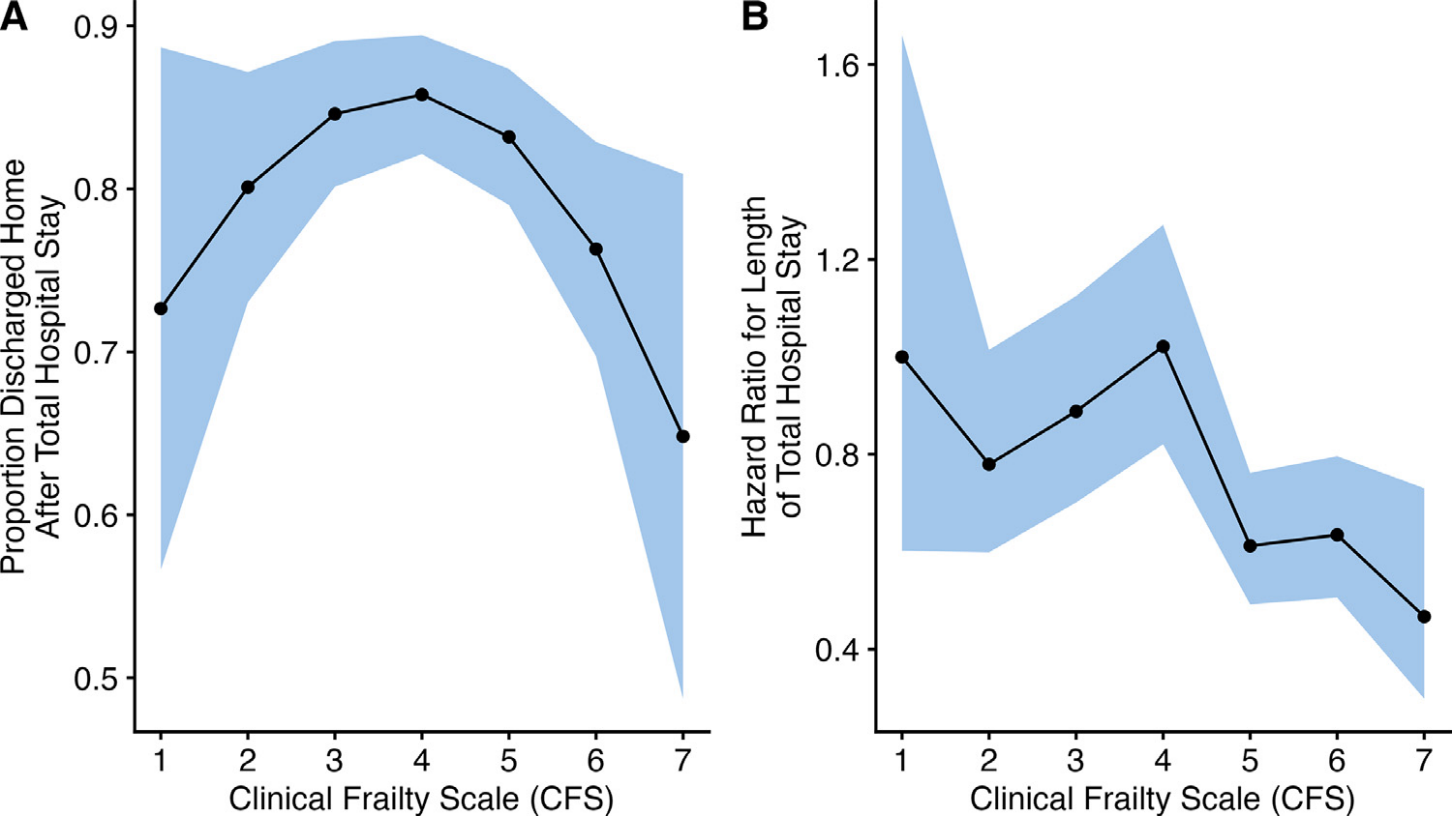
A. Proportion (95 % CI) of patients discharged home after the total (includes subacute inpatient) hospital stay across Clinical Frailty Scale scores (CFS 1 to 7). B. Hazard ratio (95 % CI) for total hospital length of stay across Clinical Frailty Scale scores (CFS 1 to 7). Abbreviations: CFS, Clinical Frailty Scale.

The total in-hospital mortality rate was four percent (*n* = 27; eTable 2). Most patients were discharged home at the end of their total hospital stay (*n* = 535, 82 %) with 11 % (*n* = 71) transferred to another health service and 7 % (*n* = 47) discharged to residential care (Fig. 2 and eTable 11). Patients with CFS scores between three and six had the greatest chance of being discharged home at the total hospital discharge timepoint (includes inpatient subacute stay at the health service if applicable; *p* = 0.027 overall effect, Fig. 4A and eTable 12). While most patients with the lowest CFS scores were discharged home (CFS 1, 67 % (*n* = 10) and CFS 2, 78 % (*n* = 50); eTable 11), these patients had proportionately the highest rates of inpatient transfers to a different health service (CFS 1, 27 % (*n* = 4) and CFS 2, 17 % (*n* = 11); eTable 11). While most patients with the highest CFS scores were discharged home (CFS 6, 71 % (*n* = 65) and CFS 7, 59 % (*n* = 10); eTable 11) these patients had proportionately more patients discharged to residential care (CFS 6, 10 % (*n* = 9) and CFS 7, 18 % (*n* = 3); eTable 11). The difference in proportion of patients discharged home between those who were and were not frail was not statistically significant (*p* = 0.139), with 79.1 % (74.2, 84.0) of frail patients and 84.0 % (80.3, 87.7) of not frail patients discharged home.

### 3.8 Total hospital length of stay

The median [IQR] total hospital length of stay was 22 [5, 47] days (eTable 13). People with lower CFS scores had a shorter length of hospital stay (*p* < 0.001 overall effect; Fig. 4B and eTable 14). The longer total hospital stay for the presence of frailty was associated with a hazard ratio of 0.62 (95 % CI: 0.52, 0.74; *p* < 0.001).

### 3.9 Sensitivity analysis

The models for change in physical function, acute hospital discharge destination, acute hospital length of stay and total hospital length of stay showed very little difference between multiple imputation analysis and the complete case analysis (eTables 15 to 18). The model for total hospital discharge destination failed to compute valid standard errors in the complete case analysis, which can be explained by small numbers in the categories for admitted specialty, resulting in “complete separation” where standard logistic regression models fail [37].

## 4 Discussion

This is the first study to examine the effect of frailty on physical function recovery in an acutely hospitalised patient cohort that received early targeted physical rehabilitation provided by physiotherapists. The key finding in this cohort of over 600 patients, was that irrespective of the degree of frailty, the mean improvement in physical function from admission to discharge exceeded the minimal clinically important difference of 5.8 points on the mILOA. Greatest improvements in physical function were observed in patients with lower degrees of frailty (CFS one to six) with a portion of patients with severe frailty (CFS 7) not making clinically meaningful gains following an Early Rehabilitation program. The mean improvement in physical function and probabilities for discharge home from the acute hospital followed a consistent and similar trend with a steady decline across CFS scores that fell steeply from CFS four to seven. Future research to explore the link between predictions for changes in physical function during hospitalisation and discharge home in similar cohorts is needed and would be of great clinical utility.

The Early Rehabilitation cohort was not specific to a hospital ward or unit. Despite this, our findings align with observational studies of a physiotherapy service for a geriatric medicine ward that examined physical recovery trajectories with the CFS [6,38]. As with our study, the CFS provided meaningful characterisation of physical recovery where better trajectories were observed in those with less frailty. Despite a similar sample size to our study, previous reports collapsed CFS categories into severe, moderate or no frailty or clustered mobility independence as low, intermediate or high [6,38]. Our findings would suggest reducing the granularity of the CFS may mask physical function recovery trajectories. Therefore, while we observed a split at CFS score of four we do not recommend prognostication based on a simple categorization of “frail” or “not frail”. The clinical ramifications of this approach can be significant where interventions that could alter the recovery trajectory are prematurely disregarded based on frailty status. For example, unlike the vulnerable (CFS 4) and not frail (CFS 1 to 3), no change was observed in the mILOA (assessed by physiotherapists) from admission to discharge for patients admitted to a trauma ward classified as frail (CFS > 4), lumping patients with mild, moderate, severe and very severe frailty and terminal illness [39]. In contrast, the findings of our study suggest there are patients with frailty who experience clinically important improvements in physical function from admission to discharge following an early rehabilitation program and provides a future opportunity to evaluate different approaches to physical rehabilitation in more discrete patient groups based on degrees of frailty.

The CFS is associated with hospital discharge destination in other studies [3]. In our study patients who were frail were less likely to return home after the acute hospital stay compared to their non-frail peers. The relationship between frailty and acute hospital length of stay was not significant. The longer total hospital stay (includes inpatient subacute stay at the health service if applicable) associated with increasing frailty observed in our study is well-described [23,40]. A limitation for the total hospital length of stay calculation was we were not able to include time spent as an inpatient after transfer to another health service. For the total hospital stay timepoint, patients with CFS scores from three to six had the greatest chance of discharge home. Patients with the lowest CFS scores (while most were discharged home, eTable 11) had proportionally the highest rates of transfer to another health service and patients with the highest CFS scores (while most were discharged home, eTable 11) had proportionally the highest rates of discharge to residential care. It is hypothesized that, especially for patients with the lowest CFS scores, with the greater time elapsed from the acute hospital time point (and more distance from the Early Rehabilitation intervention) there were extraneous factors not captured in our dataset that impacted the decision making of health professionals and logistics of discharge destination for the secondary outcomes of the total hospital stay timepoint. The primary aim of the study was to examine the effect of frailty on physical function and we considered only relevant patient and therapist goals in this domain. Patients may have remained in hospital or required rehabilitation for other reasons. Ideally early rehabilitation would be inclusive of a multidisciplinary approach to care so the relationship of frailty, recovery trajectory and other patient outcomes could be more comprehensively understood.

### 4.1 Limitations

First, the CFS was selected for frailty screening as the most widely used measure in the hospital setting and consistency in scoring agreement [3,32]. The CFS has been validated for people aged 65 and over [19]. Approximately 20 % of patients under 65 years were screened for frailty as no age limit was applied to the Early Rehabilitation cohort. We do not believe this younger sub-group materially affects our findings. The CFS is increasingly being used in younger populations as vulnerable groups are at risk of earlier frailty onset, for example people with multimorbidity [41].

Second, the study used data collected in routine clinical practice, and as such missingness of measures was expected and did occur. There is little however, to suggest it impacted the results. Only one patient had a missing frailty score, and physical function was assessed at the beginning and end of the Early Rehabilitation program in 78 % of patients. Overall, the statistical modelling showed very little difference between the multiple imputation (primary analysis) and the complete case analysis (sensitivity analysis).

Third, confounders for comorbidities and patient acuity were not included due to the limitations of the hospital administrative data. A challenge of hospital administrative data is capturing pre-existing chronic conditions of patients at the time of admission to hospital. While the International Classification of Diseases can be used to measure comorbidities (e.g. Charlson Comorbidity Index [42]) historical data are needed as scoring is labour intensive and therefore not available at hospital admission. Only half of patients who received Early Rehabilitation had a prior admission to the health service preventing the inclusion of comorbidities as a confounder in the analyses. Despite this, numerous covariates were used to adjust for potential confounding based on well-known links with frailty and the outcomes of interest (i.e. age, sex, socioeconomic status, prior residence, emergency admission and admitting specialty) [43].

Fourth, while information on premorbid mobility was obtained in the physiotherapy standardised assessment (Fig. 2) and recorded as free text in clinical notes; premorbid physical function was not assessed with the mILOA prohibiting comparison to physical function assessed at discharge.

Fifth, it is not currently feasible to describe what each patient did in their Early Rehabilitation program as the information is recorded as free text in clinical notes and would involve manual data extraction of a median [IQR] six [5, 9] Early Rehabilitation sessions for 680 patients equating to more than 4000 data points. Emerging techniques such as natural language processing (as they become more accessible) would provide a much-needed solution to examine the mediating effect of Early Rehabilitation on frailty for physical function outcomes in future research.

Last, the impact of the survivor bias is minimal where the in-hospital mortality rate was very low at less than one percent for the acute stay and four percent for the total hospital stay and is unlikely to have influenced the results.

### 4.2 Implications for clinical practice and future research

On average, patients along the continuum of frailty from very fit to severely frail experienced clinically important improvements in physical function following an acute inpatient physiotherapy early rehabilitation program. However, there was a portion of patients with severe frailty who did not make clinically meaningful gains in physical function following the Early Rehabilitation program. Patients who were frail had poorer hospital-based outcomes with longer acute hospital length of stay and reduced likelihood of direct discharge home. Despite this, on average patients who were frail experienced clinically significant improvements in physical function. In the absence of a control group, we hypothesise the physical function improvements observed in patients with frailty may not have occurred without early rehabilitation intervention; aligning with recent research on the effect of early allied health intervention for people admitted to hospital with frailty [44]. Patients admitted to hospital with frailty (CFS > 4), especially mild and moderate frailty (CFS 5 and 6), may represent a patient subgroup for targeted interventions in future research to alter their recovery trajectory. In the interim, in accordance with clinical guidelines we recommend health professionals screen adults admitted to hospital for frailty [14]. We also recommend providing, when indicated, goal-based patient-centred personalised exercise and physical rehabilitation programs during acute hospitalisation to address recently acquired mobility or physical function limitations.

Validation of the findings of this single-site observational study in another cohort is needed to provide health professionals with predictions of physical function recovery across the continuum of frailty during hospitalisation. Coupling physical function recovery predictions with data on the receipt of interventions during the acute hospital stay would facilitate investigations into rehabilitation dose-response across the frailty continuum. Such research would provide valuable prognostic information to support health professionals when making rapid and complex decisions about providing rehabilitation in hospital. At a health system level, research on personalized physical rehabilitation approaches to improve physical function recovery trajectory could in turn inform the implementation of targeted clinical pathways based on frailty and other characteristics identified early in patients’ acute hospital admission to deliver more efficient and effective health care.

## 5 Conclusion

Important clinical trends emerged in this Early Rehabilitation cohort of over 600 patients. On average, improvements in physical function from admission to discharge were clinically significant for patients irrespective of frailty status. However, there was a portion of patients with severe frailty who did not make clinically meaningful gains in physical function following Early Rehabilitation. The mean improvement in physical function and predicted probabilities for discharge home at acute hospital discharge were associated with a steady decline across frailty scores that fell steeply from CFS four to seven. Future research to validate findings from this Early Rehabilitation cohort is needed to predict recovery trajectories before implementation into clinical practice. In the interim, we recommend health professionals screen adults admitted to hospital for frailty and, when indicated, provide personalized exercise and physical rehabilitation programs to address recently acquired mobility or physical function limitations.

## CRediT authorship contribution statement

**Jennifer R A Jones:** Writing – original draft, Visualization, Supervision, Resources, Project administration, Methodology, Investigation, Funding acquisition, Formal analysis, Data curation, Conceptualization. **Sue Berney:** Writing – original draft, Supervision, Methodology, Funding acquisition, Conceptualization. **Chris Michael:** Writing – review & editing, Software, Data curation. **Tessa O’Dea:** Writing – review & editing, Methodology, Investigation, Funding acquisition, Conceptualization. **Joleen W Rose:** Writing – review & editing, Software, Methodology, Data curation. **Talia Clohessy:** Writing – review & editing, Visualization, Investigation, Data curation. **Stacey Haughton:** Writing – review & editing, Visualization, Investigation, Data curation. **Rebekah McGaw:** Writing – review & editing, Visualization, Investigation, Data curation. **Cameron Patrick:** Writing – original draft, Formal analysis. **Mark Hindson:** Writing – review & editing, Methodology, Investigation, Conceptualization. **Sharae Theisinger:** Writing – review & editing, Methodology, Investigation, Conceptualization. **Elena Gerstman:** Writing – review & editing, Methodology. **Rebecca Morris:** Writing – review & editing, Methodology. **Lucy Gao:** Writing – review & editing, Visualization, Investigation, Data curation. **David J Berlowitz:** Writing – original draft, Supervision, Resources, Methodology, Funding acquisition, Conceptualization.

## Declaration of interests

The authors declare the following financial interests/personal relationships which may be considered as potential competing interests:

Jennifer Jones reports financial support was provided by Victorian Department of Health. Sue Berney reports financial support was provided by Victorian Department of Health. Tessa O’Dea reports financial support was provided by Victorian Department of Health. David Berlowitz reports financial support was provided by Victorian Department of Health. Jennifer Jones reports financial support was provided by The University of Melbourne. If there are other authors, they declare that they have no known competing financial interests or personal relationships that could have appeared to influence the work reported in this paper.

## Appendix Supplementary materials

Image, application 1

Image, application 2
