# Supplementary material for: Association between frailty and physical function recovery of people who received physiotherapy early rehabilitation during acute hospitalisation: An observational cohort study
Source: J Frailty Aging. 2025 May 16;14(3):100052. doi: 10.1016/j.tjfa.2025.100052 (PMC12462523; doi:10.1016/j.tjfa.2025.100052)
Supplement: Supplementary file 1 [file mmc1.docx]

**Supplementary Material**

**Statistical Analysis Additional Information**

Generalised additive models allow for a flexible nonlinear relationship between explanatory variables and the outcome, without requiring an explicit functional form of the relationship to be specified, and placing a penalty on the nonlinearity of the functional relationship to avoid overfitting. The multivariate imputation by chained equations ‘mice’ package in R as used to generate 25 imputed datasets using predictive mean matching, with the following auxiliary variables which had no missing data used as predictors for the imputation: age, sex, socioeconomic disadvantage, prior residence, emergency admission, admitting specialty, length of acute hospital stay, and length of total hospital stay.^31^

eTable 1: Characteristics Survivors and Deceased for the Acute Hospital Stay

| Characteristics and Outcomes | Survivors  (n = 674) | Deceased  (n = 6) |
| --- | --- | --- |
| Age, years | 75 [66, 83] | 84 [70, 90] |
| Aged 65 and over | 524 (78) | 5 (83) |
| Sex |  |  |
| Female | 392 (58) | 2 (33) |
| Male | 282 (42) | 4 (67) |
| Prior Residence |  |  |
| Home with others | 464 (69) | 4 (67) |
| Home alone | 210 (31) | 2 (33) |
| Socioeconomic Advantage and Disadvantage | 8 [5, 9] | 5 [2, 6] |
| Socioeconomic Disadvantage | 8 [4, 9] | 4 [2, 6] |
| Clinical Frailty Scale* | 4 [3, 5] | 5 [3, 5] |
| Presence or absence of Frailty* |  |  |
| Not frail, Clinical Frailty Scale ≤ 4 | 411 (61) | 3 (50) |
| Frail, Clinical Frailty Scale > 4 | 262 (39) | 3 (50) |
| Admission mILOA** | 24 [19, 30] | 26 [21, 30] |
| Emergency admission | 354 (53) | 5 (83) |
| Admitting Specialty |  |  |
| Orthopaedics | 407 (60) | 1 (17) |
| General Medicine | 98 (15) | 0 (0) |
| Stroke, Neurology and Neurosurgery | 49 (7) | 1 (17) |
| Renal, Vascular and Urology | 31 (5) | 0 (0) |
| Haematology and Oncology | 24 (4) | 1 (17) |
| Respiratory, Cardiology, Cardiac and Thoracic Surgery | 24 (4) | 0 (0) |
| Hepatobiliary and Transplant | 18 (3) | 0 (0) |
| Other | 13 (2) | 2 (33) |
| Gastroenterology and Colorectal | 10 (1) | 1 (17) |

Continuous data are presented as median [IQR] and categorical data are presented as n (%). Physical function was measured at commencement of the Early Rehabilitation program with the modified Iowa Level of Assistance Scale (mILOA). Measures of socioeconomic status were derived from residential post codes for the Index of Relative Socio-Economic Disadvantage and the Index of Relative Socio-Economic Advantage and Disadvantage.

*Missing Clinical Frailty Scale for one survivor.

**Missing physical function data for 19 survivors.

eTable 2: Characteristics of Survivors and Deceased for the Total Hospital Stay

| Characteristics and Outcomes | Survivors  (n = 653) | Deceased  (n = 27) |
| --- | --- | --- |
| Age, years | 75 [66, 83] | 86 [76, 92] |
| Aged 65 and over | 504 (77) | 25 (93) |
| Sex |  |  |
| Female | 383 (59) | 11 (41) |
| Male | 270 (41) | 16 (59) |
| Prior Residence |  |  |
| Home with others | 452 (69) | 16 (59) |
| Home alone | 201 (31) | 11 (41) |
| Socioeconomic Advantage and Disadvantage | 8 [5, 9] | 6 [4, 9] |
| Socioeconomic Disadvantage | 8 [5, 9] | 5 [3, 9] |
| Clinical Frailty Scale* | 4 [3, 5] | 5 [4, 6] |
| Presence or absence of Frailty* |  |  |
| Not frail, Clinical Frailty Scale ≤ 4 | 405 (62) | 9 (33) |
| Frail, Clinical Frailty Scale > 4 | 247 (38) | 18 (67) |
| Admission mILOA** | 24 [19, 30] | 26 [20, 33] |
| Emergency Admission | 333 (51) | 26 (96) |
| Admitting Specialty |  |  |
| Orthopaedics | 403 (62) | 5 (19) |
| General Medicine | 89 (14) | 9 (33) |
| Stroke, Neurology and Neurosurgery | 47 (7) | 3 (11) |
| Renal, Vascular and Urology | 30 (5) | 1 (4) |
| Haematology and Oncology | 21 (3) | 4 (15) |
| Respiratory, Cardiology, Cardiac and Thoracic Surgery | 23 (4) | 1 (4) |
| Hepatobiliary and Transplant | 18 (3) | 0 (0) |
| Other | 13 (2) | 2 (7) |
| Gastroenterology and Colorectal | 9 (1) | 2 (7) |
| Acute hospital length of stay, days | 9 [5, 20] | 20 [14, 38] |
| Total hospital length of stay, days | 22 [5, 47] | 40 [30, 49] |

Continuous data are presented as median [IQR] and categorical data are presented as n (%). Physical function was measured at commencement of the Early Rehabilitation program with the modified Iowa Level of Assistance Scale (mILOA). Measures of socioeconomic status were derived from residential post codes for the Index of Relative Socio-Economic Disadvantage and the Index of Relative Socio-Economic Advantage and Disadvantage.

*Missing Clinical Frailty Scale for one survivor.

**Missing physical function data for 16 survivors and three deceased.

eTable 3: Characteristics of Patients with Paired Physical Function Data vs Missing Data

| Characteristics and Outcomes | Paired Data  (n = 529) | Missing Data  (n = 145) | p-value |
| --- | --- | --- | --- |
| Age, years | 76 [67, 83] | 74 [64, 81] | 0.2 |
| Aged 65 and over | 420 (79) | 104 (72) | 0.064 |
| Sex |  |  | 0.9 |
| Female | 309 (58) | 83 (57) |  |
| Male | 220 (42) | 62 (43) |  |
| Prior Residence |  |  | 0.8 |
| Home with others | 366 (69) | 98 (68) |  |
| Home alone | 163 (31) | 47 (32) |  |
| Socioeconomic Advantage and Disadvantage | 8 [5, 9] | 8 [6, 9] | 0.3 |
| Socioeconomic Disadvantage | 8 [4, 9] | 8 [5, 9] | 0.3 |
| Clinical Frailty Scale* | 4 [3, 5] | 4 [3, 5] | >0.9 |
| Presence or absence of Frailty* |  |  | 0.5 |
| Not frail, Clinical Frailty Scale ≤ 4 | 327 (62) | 84 (58) |  |
| Frail, Clinical Frailty Scale > 4 | 202 (38) | 60 (42) |  |
| Emergency Admission | 277 (52) | 77 (53) | >0.9 |
| Admitting Specialty |  |  | 0.084 |
| Orthopaedics | 323 (61) | 84 (58) |  |
| General Medicine | 76 (14) | 22 (15) |  |
| Stroke, Neurology and Neurosurgery | 41 (8) | 8 (6) |  |
| Renal, Vascular and Urology | 25 (5) | 6 (4) |  |
| Haematology and Oncology | 17 (3) | 7 (5) |  |
| Respiratory, Cardiology, Cardiac and  Thoracic Surgery | 16 (3) | 8 (6) |  |
| Hepatobiliary and Transplant | 16 (3) | 2 (1) |  |
| Other | 6 (1) | 7 (5) |  |
| Gastroenterology and Colorectal | 9 (2) | 1 (1) |  |

Continuous data are presented as median [IQR] and categorical data are presented as n (%). The Kruskal-Wallis rank sum test was used for continuous data and Pearsons’ Chi-squared for categorical data. Significance was set at p < 0.05. Measures of socioeconomic status were derived from residential post codes for the Index of Relative Socio-Economic Disadvantage and the Index of Relative Socio-Economic Advantage and Disadvantage.

*Missing data for the Clinical Frailty Scale for one patient with paired physical function data.

eTable 4: Characteristics of the Cohort

| Patient Characteristics | n = 680 |
| --- | --- |
| Age, years | 75 [66, 83] |
| Aged 65 and over | 529 (78) |
| Sex |  |
| Female | 394 (58) |
| Male | 286 (42) |
| Prior Residence |  |
| Home with others | 468 (69) |
| Home alone | 212 (31) |
| Socioeconomic Advantage and Disadvantage | 8 [5, 9] |
| Socioeconomic Disadvantage | 8 [4, 9] |
| Clinical Frailty Scale | 4 [3, 5] |
| Presence or absence of Frailty* |  |
| Not frail, Clinical Frailty Scale ≤ 4 | 414 (61) |
| Frail, Clinical Frailty Scale > 4 | 265 (39) |
| Admission mILOA | 24 [19, 30] |
| Emergency Admission | 359 (53) |
| Admitting Specialty |  |
| Orthopaedics | 408 (60) |
| General Medicine | 98 (14) |
| Stroke, Neurology and Neurosurgery | 50 (7) |
| Renal, Vascular and Urology | 31 (5) |
| Haematology and Oncology | 25 (4) |
| Respiratory, Cardiology, Cardiac and Thoracic Surgery | 24 (4) |
| Hepatobiliary and Transplant | 18 (3) |
| Other | 15 (2) |
| Gastroenterology and Colorectal | 11 (2) |
| Principal Diagnosis |  |
| Musculoskeletal, Hip or Knee Replacement | 254 (37) |
| Musculoskeletal, Pelvis, Hip or Femur Trauma or Interventions | 132 (19) |
| Musculoskeletal, Other | 63 (9) |
| Other | 58 (9) |
| Digestive, Hepatobiliary, Pancreas, Endocrine, Metabolic, Kidney and Urinary Tract | 43 (6) |
| Neurology | 36 (5) |
| Stroke | 29 (4) |
| Haematology, Immunology, Oncology, Infectious Diseases or Toxicology | 28 (4) |
| Circulation | 21 (3) |
| Respiratory | 16 (2) |

Continuous data are presented as median [IQR] and categorical data are presented as n (%). Measures of socioeconomic status were derived from residential post codes for the Index of Relative Socio-Economic Disadvantage and the Index of Relative Socio-Economic Advantage and Disadvantage. Physical function was measured at commencement of the Early Rehabilitation program with the modified Iowa Level of Assistance Scale (mILOA). Primary diagnoses were classified using the Australian Refined Diagnosis Related Groups.

*Missing Clinical Frailty Scale for one patient.

**Missing physical function for 19 patients.

eTable 5: Number of Early Rehabilitation Sessions Across Frailty Scores

| Number of Early Rehabilitation Sessions | CFS 1  (n=17) | CFS 2  (n=64) | CFS 3  (n=110) | CFS 4  (n=220) | CFS 5  (n=143) | CFS 6  (n=99) | CFS 7  (n=20) | p-value |
| --- | --- | --- | --- | --- | --- | --- | --- | --- |
| Number of Sessions | 6 [4, 9] | 6 [4, 8] | 6 [5, 9] | 8 [6, 10] | 6 [5, 9] | 5 [5, 7] | 6 [4, 7] | <0.001 |

Continuous data are presented as median [IQR] and the Kruskal-Wallis rank sum test was used. Significance was set at p < 0.05.

Abbreviations: CFS, Clinical Frailty Scale.

eTable 6: Physical Function Model following Multiple Imputation

| CFS | n | Mean Difference (95%) in Physical Function | Mean (95% CI) Admission Physical Function | Mean (95% CI) Discharge Physical Function |
| --- | --- | --- | --- | --- |
| 1 | 17 | -11.2 (-13.9, -8.6) | 23.3 (18.7, 28.0) | 13.2 (7.4, 19.0) |
| 2 | 64 | -11.6 (-12.9, -10.3) | 22.4 (20.3, 24.5) | 11.6 (9.0, 14.2) |
| 3 | 110 | -11.8 (-12.8, -10.9) | 23.2 (21.9, 24.6) | 10.3 (8.4, 12.2) |
| 4 | 220 | -11.1 (-12.0, -10.3) | 22.5 (21.7, 23.3) | 9.7 (8.5, 10.9) |
| 5 | 144 | -9.6 (-10.5, -8.7) | 25.3 (24.2, 26.4) | 16.6 (14.8, 18.4) |
| 6 | 99 | -8.0 (-9.2, -6.8) | 26.1 (24.7, 27.4) | 20.4 (18.6, 22.3) |
| 7 | 20 | -6.5 (-8.9, -4.2) | 30.2 (27.8, 32.6) | 26.1 (22.5, 29.7) |

Generalised additive models were used to assess the average effect of frailty score the mean difference (95% CI) in physical function (i.e. change in modified Iowa Level of Assistance Scale score). Mean (95% CI) admission and discharge physical function are provided. Physical function was measured with the modified Iowa Level of Assistance Scale which ranges from 0 to 36 where lower scores indicate better physical function and the minimal detectable change is 5.8 points, therefore the reduction in scores from admission to discharge denotes improvement. Note that the lower limit of the change in mILOA is beyond the minimal detectable change in all but those with an admission CFS of 7.

Abbreviations: CFS, Clinical Frailty Scale.

eTable 7: Acute Hospital Discharge Destination Across Frailty Scores

| Discharge Destination | CFS 1  (n=17) | CFS 2  (n=64) | CFS 3  (n=110) | CFS 4  (n=220) | CFS 5  (n=143) | CFS 6  (n=99) | CFS 7  (n=20) |
| --- | --- | --- | --- | --- | --- | --- | --- |
| Home | 6 (35) | 31 (48) | 55 (50) | 140 (64) | 49 (34) | 16 (16) | 1 (5) |
| Inpatient Rehabilitation | 6 (35) | 26 (41) | 42 (38) | 55 (25) | 75 (52) | 71 (72) | 15 (75) |
| Hospital Transfer | 3 (18) | 7 (11) | 7 (6) | 12 (5) | 7 (5) | 6 (6) | 2 (10) |
| Other | 2 (12) | 0 (0) | 6 (5) | 13 (6) | 12 (8) | 6 (6) | 2 (10) |

Data are presented as n (%). The Kruskal-Wallis rank sum test was used for continuous data. Significance was set at p < 0.05.

eTable 8: Proportion of Patients Discharged Home at Acute Hospital Discharge Across Frailty Scores following Multiple Imputation

| CFS | n | Proportion Discharged Home (95% CI) |
| --- | --- | --- |
| 1 | 17 | 0.510 (0.363, 0.657) |
| 2 | 64 | 0.509 (0.431, 0.588) |
| 3 | 110 | 0.501 (0.448, 0.555) |
| 4 | 220 | 0.477 (0.433, 0.521) |
| 5 | 144 | 0.402 (0.354, 0.451) |
| 6 | 99 | 0.310 (0.234, 0.387) |
| 7 | 20 | 0.217 (0.086, 0.348) |

Proportion (95% CI) of patients discharged home at acute hospital discharge across frailty scores are provided. Generalised additive models were used to assess the average effect of frailty score on discharge home, a binomial response distribution and logit link function were used, similar to a standard logistic regression model. Abbreviations: CFS, Clinical Frailty Scale.

eTable 9: Acute Hospital Length of Stay Across Frailty Scores

| Acute Hospital Length of Stay | CFS 1  (n=17) | CFS 2  (n=64) | CFS 3  (n=110) | CFS 4  (n=220) | CFS 5  (n=143) | CFS 6  (n=99) | CFS 7  (n=20) | p-value |
| --- | --- | --- | --- | --- | --- | --- | --- | --- |
| Length of Stay, Days | 15 [6, 23] | 14 [5, 29] | 8 [4, 23] | 6 [4, 13] | 10 [6, 20] | 14 [10, 22] | 22 [9, 27] | <0.001 |

Data are presented as median [IQR]. The Kruskal-Wallis rank sum test was used for continuous data. Significance was set at p < 0.05. Abbreviations: CFS, Clinical Frailty Scale.

eTable 10: Hazard Ratios for Acute Hospital Length of Stay Across Frailty Scores

| CFS | n | Hazard Ratio (95% CI) |
| --- | --- | --- |
| 1 | 17 | 1.00 (0.78, 1.29) |
| 2 | 64 | 0.98 (0.80, 1.20) |
| 3 | 110 | 0.96 (0.81, 1.14) |
| 4 | 220 | 0.94 (0.81, 1.10) |
| 5 | 144 | 0.92 (0.78, 1.07) |
| 6 | 99 | 0.89 (0.74, 1.07) |
| 7 | 20 | 0.86 (0.68, 1.09) |

Hazard ratios (95% CI) for acute hospital length of stay across frailty scores, normalised so CFS of 1 had a HR of 1.00. The length of stay outcomes were modelled as time-to-event using a Cox proportional hazards generalised additive model. Abbreviations: CFS, Clinical Frailty Scale.

eTable 11: Discharge Destination after Total Hospital Stay Across Frailty Scores

| Discharge Destination | CFS 1  (n=15) | CFS 2  (n=64) | CFS 3  (n=110) | CFS 4  (n=216) | CFS 5  (n=139) | CFS 6  (n=91) | CFS 7  (n=17) |
| --- | --- | --- | --- | --- | --- | --- | --- |
| Home | 10 (67) | 50 (78) | 93 (85) | 192 (89) | 114 (82) | 65 (71) | 10 (59) |
| Hospital Transfer | 4 (27) | 11 (17) | 13 (12) | 18 (8) | 13 (9) | 9 (10) | 3 (18) |
| Residential Care | 1 (7) | 3 (5) | 4 (4) | 6 (3) | 12 (9) | 17 (19) | 4 (24) |

Continuous data are presented n (%). Abbreviations: CFS, Clinical Frailty Scale.

eTable 12: Proportion of Patients Discharged Home after Total Hospital Stay following Multiple Imputation

| CFS | n | Proportion Discharged Home (95% CI) |
| --- | --- | --- |
| 1 | 15 | 0.727 (0.566, 0.887) |
| 2 | 64 | 0.801 (0.730, 0.872) |
| 3 | 110 | 0.846 (0.801, 0.891) |
| 4 | 216 | 0.858 (0.821, 0.894) |
| 5 | 140 | 0.832 (0.790, 0.874) |
| 6 | 91 | 0.763 (0.697, 0.829) |
| 7 | 17 | 0.648 (0.487, 0.809) |

Proportion (95% CI) of patients discharged home after total hospital stay across frailty scores. Generalised additive models were used to assess the average effect of frailty score on discharge home, a binomial response distribution and logit link function were used. Abbreviations: CFS, Clinical Frailty Scale.

eTable 13: Total Hospital Length of Stay Across Frailty Scores

| Total Hospital Length of Stay | CFS 1  (n=15) | CFS 2  (n=64) | CFS 3  (n=110) | CFS 4  (n=216) | CFS 5  (n=139) | CFS 6  (n=91) | CFS 7  (n=17) | p-value |
| --- | --- | --- | --- | --- | --- | --- | --- | --- |
| Length of Stay, Days | 15 [4, 69] | 25 [5, 66] | 17 [4, 45] | 7 [4, 30] | 33 [10, 54] | 39 [26, 55] | 45 [35, 73] | <0.001 |

Continuous data are presented as median [IQR]. The Kruskal-Wallis rank sum test was used for continuous data. Significance was set at p < 0.05. Abbreviations: CFS, Clinical Frailty Scale

eTable 14: Hazard Ratios for Total Hospital Length of Stay Across Frailty Scores following Multiple Imputation

| CFS | n | Hazard Ratio (95% CI) |
| --- | --- | --- |
| 1 | 15 | 1.00 (0.60, 1.66) |
| 2 | 64 | 0.78 (0.60, 1.01) |
| 3 | 110 | 0.89 (0.70, 1.12) |
| 4 | 216 | 1.02 (0.82, 1.27) |
| 5 | 140 | 0.61 (0.49, 0.76) |
| 6 | 91 | 0.64 (0.51, 0.80) |
| 7 | 17 | 0.47 (0.30, 0.73) |

Hazard ratios (95% CI) for total hospital length of stay across frailty scores, normalised so CFS of 1 had a HR of 1.00. The length of stay outcomes were modelled as time-to-event using a Cox proportional hazards generalised additive model.

Abbreviations: CFS, Clinical Frailty Scale.

eTable 15: Physical Function Model for the Complete Case Analysis

| CFS | n | Mean Difference in Physical Function (95% CI) | Mean (95% CI) Admission Physical Function | Mean (95% CI) Discharge Physical Function |
| --- | --- | --- | --- | --- |
| 1 | 9 | -11.3 (-14.2, -8.5) | 24.0 (16.5, 31.5) | 14.4 (6.5, 22.4) |
| 2 | 51 | -11.8 (-13.2, -10.4) | 23.8 (21.6, 26.0) | 12.9 (9.9, 15.9) |
| 3 | 92 | -12.1 (-13.1, -11.2) | 22.7 (21.2, 24.2) | 9.4 (7.5, 11.2) |
| 4 | 175 | -11.4 (-12.2, -10.6) | 22.5 (21.6, 23.4) | 9.2 (8.0, 10.5) |
| 5 | 104 | -9.8 (-10.7, -9.0) | 25.5 (24.3, 26.8) | 16.8 (14.8, 18.8) |
| 6 | 84 | -8.4 (-9.6, -7.2) | 25.9 (24.4, 27.4) | 20.2 (18.3, 22.1) |
| 7 | 14 | -7.2 (-9.6, -4.7) | 30.4 (27.4, 33.3) | 25.4 (21.4, 29.4) |

Generalised additive models were used to assess the average effect of frailty score on the mean difference (95% CI) in physical function (i.e. change in modified Iowa Level of Assistance Scale score). Mean (95% CI) physical function at beginning and end of the Early Rehabilitation program are provided. Physical function was measured with the modified Iowa Level of Assistance Scale which ranges from 0 to 36 where lower scores indicate better physical function and the minimal detectable change is 5.8 points, therefore the reduction in scores from beginning to end denotes improvement.

Abbreviations: CFS, Clinical Frailty Scale.

eTable 16: Proportion of Patients Discharged Home at Acute Hospital Discharge Across Frailty Scores for the Complete Case Analysis

| CFS | n | Proportion Discharged Home (95% CI) |
| --- | --- | --- |
| 1 | 9 | 0.424 (0.230, 0.619) |
| 2 | 51 | 0.485 (0.392, 0.579) |
| 3 | 92 | 0.530 (0.468, 0.592) |
| 4 | 175 | 0.519 (0.467, 0.570) |
| 5 | 104 | 0.432 (0.375, 0.489) |
| 6 | 84 | 0.340 (0.253, 0.427) |
| 7 | 14 | 0.246 (0.078, 0.414) |

Proportion (95% CI) of patients discharged home at acute hospital discharge across frailty scores. Generalised additive models were used to assess the average effect of frailty score on discharge home, a binomial response distribution and logit link function were used.

Abbreviations: CFS, Clinical Frailty Scale.

eTable 17: Hazard Ratios for Acute Hospital Length of Stay Across Frailty Scores for the Complete Case Analysis

| CFS | n | Hazard Ratio (95% CI) |
| --- | --- | --- |
| 1 | 9 | 1.00 (0.70, 1.47) |
| 2 | 51 | 1.12 (0.86, 1.45 |
| 3 | 92 | 1.23 (0.99, 1.54) |
| 4 | 175 | 1.29 (1.04, 1.61) |
| 5 | 104 | 1.27 (1.03, 1.57) |
| 6 | 84 | 1.22 (0.98, 1.53) |
| 7 | 14 | 1.17 (0.84, 1.63) |

Hazard ratios (95% CI) for acute hospital length of stay across frailty scores. The length of stay outcomes were modelled as time-to-event using a Cox proportional hazards generalised additive model.

Abbreviations: CFS, Clinical Frailty Scale.

eTable 18: Hazard Ratios for Total Hospital Length of Stay for the Complete Case Analysis

| CFS | n | Hazard Ratio (95% CI) |
| --- | --- | --- |
| 1 | 8 | 1.00 (0.49, 2.04) |
| 2 | 51 | 0.78 (0.58, 1.06) |
| 3 | 92 | 1.08 (0.83, 1.41) |
| 4 | 172 | 1.17 (0.91, 1.50) |
| 5 | 101 | 0.77 (0.60, 0.99) |
| 6 | 77 | 0.73 (0.56, 0.95) |
| 7 | 12 | 0.57 (0.34, 0.95) |

Hazard ratios (95% CI) for total hospital length of stay across frailty scores.The length of stay outcomes were modelled as time-to-event using a Cox proportional hazards generalised additive model.

Abbreviations: CFS, Clinical Frailty Scale.
